# Attention dysfunction of postoperative patients with glioma

**DOI:** 10.1186/1477-7819-12-317

**Published:** 2014-10-15

**Authors:** Dazhao Fang, Jian Jiang, Xiaoyang Sun, Weijie Wang, Nan Dong, Xianhua Fu, Cong Pang, Xingui Chen, Lianshu Ding

**Affiliations:** Department of Neurosurgery, Huai’an First People’s Hospital, Nanjing Medical University, 6 Beijing Road West, Huai’an, Jiangsu 223300 P. R. China; Department of Neurology, the First Affiliated Hospital of Anhui Medical University, 81 Meishan Road, Hefei, Anhui 230032 P. R. China

**Keywords:** Glioma, Attention deficit, Care

## Abstract

**Background:**

Attention dysfunction has been observed among many kinds of nervous system diseases, including glioma. This study aimed to investigate the correlation between glioma localization, malignancy, postoperative recovery time and attention deficit.

**Methods:**

A total of 45 patients with glioma who underwent surgical resection and 18 healthy volunteers were enrolled. The attention network test, digital span test, color trail test II and Stroop test were used to detect the characteristics of attention deficit.

**Results:**

Orientation network dysfunction was detected in the parietal lobe tumor group, and execution network deficit was detected in both the frontal and parietal lobe groups, while no significant difference was detected in the temporal lobe group compared to healthy controls. The high-grade glioma group (grade III-IV) exhibited more serious functional impairment than the low-grade group (grade I-II). No significant correlation was observed between postoperative recovery time and attention impairment.

**Conclusions:**

High-grade glioma patients suffer more severe attention impairment. In addition, the frontal and parietal lobe glioma patients suffer attention dysfunction in dissimilar manner. These findings will provide important guidance on the care of glioma patients after therapy.

## Background

Glioma is the most common malignant intracranial tumor in adults, with a poor prognosis [1]. Great effort has been made to improve the clinical outcome, especially to prolong the postoperative survival of glioma patients. However, the impairment of cognitive function after operation which obviously affects the quality of life seems frequently neglected in the current literature [2]. As a major component of the cognitive system, attention is an important aspect of brain or mental activities [3]. Posner and Petersen claimed that attention consists of three different networks (alerting, orienting and executing), based on neuronal function and neuronal anatomy, and concluded that the impairment of attention components is different in different injured brain regions [3]. The reduction in attention functionality has emerged as one of the most common postoperative complications of patients with glioma. In the present study, we investigated the attention characteristics of 45 postoperative patients with glioma who discharged from our hospital between 2010 to 2012, as well as 18 healthy controls, and analyzed the relationship between tumor localization, malignancy, postoperative recovery time and attention dysfunction.

## Methods

### Subjects

This study was approved by the ethics committee of Huai’an First People’s Hospital and all participants signed informed consent. The participants were divided into two groups. The tumor group consisted of 45 right-handed patients with glioma who underwent surgical treatment in our hospital between 2010 and 2012. The inclusion criteria were as follows: (1) diagnosed with glioma by magnetic resonance imaging scan and confirmed by pathologic examination; (2) achieved a gross total resection under microscopy; (3) mini-mental state examination (MMSE) score ≥24; (4) no deficit in visual, auditory, understanding and physical activity; (5) the absence of other significant neurological and psychiatric disorders; (6) education background enough to guarantee the understanding of task. All patients achieved gross total resection and they received no additional therapy after surgery. The control group consisted of 18 right-handed healthy controls who were medical workers and patients’ family members with MMSE scores ≥24.

### Attention network test

The attention network test was based on the attention network theory proposed by Posner and Petersen [3] and has been used to assess the attention work for any pathologic change in different brain regions. The characteristics of alerting, orienting and execution network were evaluated by the attention network test as previously described [4]. Subjects were required to respond by clicking on the directional buttons when a stimulus emerged on the screen. The pattern of the cue was changed to check the alerting and orienting function of the attention network. The characteristics of different components of the attention network were evaluated via the response time (RT) of each operation recorded automatically by the computer. Alerting effect = RT_no cue_ - RT_cue_; orienting effect = RT_center cue_ - RT_spatial cue_; execution effect = RT_incongruent_ - RT_congruent_. A higher alerting or orienting effect score indicated better alerting or orienting function, while a higher execution effect score indicated more serious execution deficit, because a longer time was needed to execute correctly upon the incongruent cue.

### Digital span test

The digital span test was used to assess the ability of focusing the mind, anti-jamming and transient memory. The subjects were asked to repeat digits immediately after the investigator. The number of digits increased and the highest number was taken as the score.

### Color trail test II

The color trail test II is frequently utilized to assess the capability of attention conversion. Numbers were placed in circles with the background colors of red or yellow, and subjects were required to connect the numbers in numerical order. Completion time was recorded as the index of attention conversion - the longer the time, the lower the efficiency of attention conversion.

### Stroop test

The Stroop test was used to illustrate the efficiency of focusing, selective attention and execution. There pieces of card were used: the first one showed some dots printed in four different colors (red, green, blue and yellow); the second card showed the characters printed in four different colors described above (no relationship between the characters and the color); the third card illustrated the characters with the meaning of the four different colors previously described and printed in the color corresponding to the meaning. The subjects were asked to read the color of the dots or characters regardless of the meaning of characters. The completion time of the different cards were respectively recorded. The final word-color interference value = CT_card 3_ - CT_card 2_, where CT is the completion time - the bigger the value, the lower the efficiency of attention.

### Statistical analysis

The data are presented as mean ± standard deviation and were analyzed by SPSS 18.0 software (SPSS Inc., Chicago, IL, USA). The differences between groups were examined using the Mann–Whitney U-test. The level of significance was set at *P* <0.05 for two-tailed tests.

## Results

### Characteristics of the subjects

Among the 45 glioma cases enrolled in this study, 15 gliomas were located in the frontal lobe, 12 in the parietal lobe and 18 in the temporal lobe; 13 cases were pathologically diagnosed as high-grade glioma (grade III-IV) and 32 cases as low-grade glioma (grade I-II) according to the World Health Organization diagnostic criteria. All the patients were categorized into two groups based on postoperative recovery time (28 cases recovered within 12 months, and 17 cases recovered after 12 months). No significant differences in the age, gender, education level and MMSE score were detected among the different groups (Table 1).

**Table 1:** **The characteristics of glioma patients and controls**

	Cases (n)	Gender (male/female)	Age (years)	Education level (years)	MMSE
Frontal lobe	15	10/5	43.13 ± 7.53	10.20 ± 3.43	26.53 ± 1.25
Parietal lobe	12	8/4	41.17 ± 8.76	10.50 ± 4.17	27.50 ± 1.31
Temporal lobe	18	10/8	42.94 ± 8.11	9.44 ± 3.94	27.71 ± 1.33
High-grade (grade III-IV)	13	9/4	44.15 ± 9.46	9.46 ± 3.31	26.77 ± 1.09
Low grade (grade I-II)	32	19/13	41.88 ± 7.33	10.41 ± 4.27	27.56 ± 1.32
Postoperative recovery (≤12 months)	28	17/11	42.71 ± 7.31	10.04 ± 3.63	27.61 ± 1.32
Postoperative recovery (>12 months)	17	11/6	42.24 ± 9.16	10.29 ± 4.67	27.71 ± 1.26
Control	18	10/8	40.37 ± 10.7	9.38 ± 5.72	28.43 ± 1.67

Clinical cases were classified into different groups based on distinct tumor localization, malignancy and postoperative recovery time, respectively. No significant differences in age, gender, education level and score of mini-mental state examination (MMSE) were detected among the different groups (*P* >0.05).

### Tumor localization is related to attention impairment

Compared with the control group, the patients with gliomas in the frontal lobe exhibited increased execution response time (Z = -2.531; *P* <0.05), while those in the parietal lobe group exhibited significant abnormal orienting and execution response time (Z = 2.159 and -2.498, respectively; *P* <0.05). However, there were no significant differences in orienting and execution response time between the temporal and control groups. Moreover, the mean response time among the different tumor groups and the healthy control group was similar (Table 2).

**Table 2:** **Association of tumor localization and attention of postoperative glioma patients**

	Cases (n)	Alerting network	Orienting network	Execution network	Mean response time (ms)
Frontal lobe	15	39.7 ± 23.8	74.9 ± 34.2	134.3 ± 42.4*	755.2 ± 131.2
Parietal lobe	12	36.8 ± 22.9	48.4 ± 22.1*	133.2 ± 35.6*	725.0 ± 113.5
Temporal lobe	18	32.1 ± 21.6	61.3 ± 17.8	117.0 ± 34.8	693.7 ± 134.5
Controls	18	36.9 ± 20.6	65.4 ± 23.4	91.8 ± 41.7	695.3 ± 106.7

*P <0.05, compare with the control group.

### Tumor malignancy is related to attention impairment

The efficiency of focusing, attention conversion and selective attention were evaluated by the digital span test, color trail test II and Stroop test. A reduction in the digital span, and increased color trail test II and Stroop test times were obvious in the high-grade glioma group compared to the low-grade group. These data indicated that the patients with high-grade glioma had more severe impairment of attention. However, we failed to detect any difference in attention impairment between the short-term and long-term recovery time groups (Table 3).

**Table 3:** **Association of tumor malignancy and postoperative recovery time and attention of postoperative glioma patients**

	Cases (n)	Digital span	Color trail test II (s)	Stroop test (s)
Frontal lobe	15	7.22 ± 1.20	130.32 ± 20.53	10.42 ± 1.53
Parietal lobe	12	6.12 ± 0.93	132.76 ± 22.14	11.28 ± 1.97
Temporal lobe	18	6.98 ± 1.01	123.70 ± 20.88	10.26 ± 1.76
High grade (grade III-IV)	13	5.76 ± 0.83*	141.48 ± 18.06*	9.20 ± 1.13*
Low grade (grade I-II)	32	6.42 ± 0.80	115.52 ± 25.79	8.15 ± 1.29
Postoperative recovery time (≤12 months)	28	6.16 ± 1.18	125.21 ± 21.75	8.27 ± 1.63
Postoperative recovery time (>12 month)	17	5.86 ± 0.82	132.12 ± 24.05	9.12 ± 1.12
Normal data for healthy Chinese	-	7.75	97.53	9.15

*P <0.05, compared to low-grade glioma.

## Discussion

The prognosis of cognitive function is a crucial element which affects the quality of life and even overall survival among postoperative glioma patients [5, 6]. Earlier studies indicated that cognitive performance may be more sensitive than computed tomography or magnetic resonance imaging scanning for the diagnosis of glioma recurrence [7, 8]. Cognitive function is now recognized as an independent prognostic factor for the survival of glioma patients, and cognitive deterioration is the first indicator of progressive disease after treatment [9].

In this study we examined the attention function in postoperative glioma patients and investigated the relationship between tumor localization, malignancy, postoperative recovery time and attention dysfunction. We observed that the different localization of the tumor has different effects on the impairment of the attention network. Among patients with glioma of the frontal lobe, the efficiency of the execution network was significantly decreased. In contrast, parietal lobe patients underwent a decline in the orienting and execution networks, while no significant difference in any attention networks was found among temporal lobe patients. These observations are in agreement with results reported recently [10]. The dorsolateral prefrontal cortex plays a crucial role in the control of cognitive function, especially the execution [11, 12]. Furthermore, the parietal lobe is supposed to affect the ability of spatial processing, and the successful completion of orienting is dependent on the bilateral inferior parietal lobe [13]. Consistent with the previous studies, we found that patients with parietal glioma showed lower efficiency of the orienting network.

In order to clarify whether the malignancy of glioma affects the impairment of attention, we divided the glioma patients into two groups: the high-grade group (grade III-IV) and the low-grade group (grade I-II). The digital span test, color trail test II and Stroop test showed that high-grade glioma patients suffered more severe deficit in attention efficiency than low-grade patients, in agreement with a previous conclusion that low-grade glioma patients performed better than high-grade patients in cognitive function [14].

Furthermore, we investigated the correlation between postoperative recovery time and attention impairment. A critical time point was set at 12 months after surgical treatment and the patients were divided into two groups accordingly. The results of the digital span test, color trail test II and Stroop test showed no significant differences between the two groups, indicating that postoperative recovery time may not be a major factor that affects the attention work. Whether a duration of 12 months is long enough to assess the effect of recovery time on attention recovery requires further investigation. In addition, it is a limitation of this study that we failed to perform the test at different time points and we would like to enroll more subjects and investigate the time effect in future.

As it is well known that patients who suffer an operation may be impaired after that operation, the effects of anesthetic should have been taken into consideration. While the administration of an anesthetic is definitely inevitable for patients who require a brain tumor surgical resection, the individuals involved in this study received the same type of anesthesia. Thus, we think that the anesthetic effect on the attention impairment of all these participants is minimal.

## Conclusions

In summary, we investigated the characteristics of attention impairment of postoperative glioma patients, and found that the high-grade glioma patients suffer more severe attention impairment. In addition, the frontal and parietal lobe glioma patients suffer attention dysfunction in a dissimilar manner. These finding will provide important guidance on the care of glioma patients after therapy.

## Consent

Written informed consent was obtained from the patients for the publication of this report.

## Abbreviations

MMSE: mini-mental state examination
RT: response time.
